# Self-assessed masticatory function and frailty in Brazilian older adults: the FIBRA Study

**DOI:** 10.11606/s1518-8787.2022056004195

**Published:** 2022-11-18

**Authors:** Clarice Santana Milagres, Luísa Helena do Nascimento Tôrres, Talita Bonato de Almeida, Anita Liberalesso Neri, José Leopoldo Ferreira Antunes, Maria da Luz Rosário de Sousa

**Affiliations:** I Faculdade São Leopoldo Mandic Departamento de Medicina Araras SP Brasil Faculdade São Leopoldo Mandic. Departamento de Medicina. Araras, SP, Brasil; II Universidade Federal de Santa Maria Departamento de Estomatologia Centro de Ciências da Saúde Santa Maria RS Brasil Universidade Federal de Santa Maria. Centro de Ciências da Saúde. Departamento de Estomatologia. Santa Maria, RS, Brasil; III Universidade Anhembi Morumbi Piracicaba SP Brasil Universidade Anhembi Morumbi. Piracicaba, SP, Brasil; IV Universidade Estadual de Campinas Faculdade de Educação Departamento de Psicologia da Educação Campinas SP Brasil Universidade Estadual de Campinas. Faculdade de Educação. Departamento de Psicologia da Educação. Campinas, SP, Brasil; V Universidade de São Paulo Faculdade de Saúde Pública Departamento de Epidemiologia São Paulo SP Brasil Universidade de São Paulo. Faculdade de Saúde Pública. Departamento de Epidemiologia. São Paulo, SP, Brasil; VI Universidade Estadual de Campinas Faculdade de Odontologia Departamento de Ciências da Saúde e Odontologia Infantil Piracicaba SP Brasil Universidade Estadual de Campinas. Faculdade de Odontologia. Departamento de Ciências da Saúde e Odontologia Infantil. Piracicaba, SP, Brasil

**Keywords:** Aged, Frail Elderly, Mastication, Patient Health Questionnaire, Diagnostic Self Evaluation

## Abstract

**OBJETIVE:**

To investigate the relationship between the masticatory function and the frailty of older people.

**METHODS:**

Exploratory cross-sectional study using secondary data from the FIBRA Project on the frailty conditions of older people living in urban areas of six Brazilian municipalities, from 2008 to 2009. The population consisted of older adults aged 65 and over with no cognitive impairment. A single session identification questionnaire and the Mini-Mental State Examination (MMSE) were applied, followed by collecting sociodemographic data and data on the participants’ self-assessment of masticatory function, general health, and oral health.

**RESULTS:**

2,341 older people (164 frail older adults), of whom 63.2% reported not having seen a dentist in the last year, and approximately 26% of them had three or more functional feeding problems. Older participants (OR = 2.88; 95%CI: 2.01–4.13), who are retired (OR = 2.31; 95%CI: 1.18–5.53), those who were depressed (OR = 2.31; 95%CI: 1.58–3.38), and those who self-assessed their general health as worse compared to others of the same age (OR = 3.91; 95%CI: 2.39–6.40) were at higher risk of frailty. Three or more problems related to the functional aspects of mastication were associated with a greater chance of frailty (OR = 2.06; 95%CI: 1.25–3.41).

**CONCLUSION:**

This study found an association between masticatory function and a greater chance of frailty among the studied population.

## INTRODUCTION

The changes in Brazil's demographic and epidemiological transitions have increased the prevalence of comorbidities and syndromes in older adults^1^. Frailty is one of the syndromes associated with aging, which is characterized by clinical manifestations such as reduced strength, fatigue, reduced walking speed, low physical activity level, and weight loss. The absence of these factors indicates robustness, whereas the presence of one or two of them suggest a high risk of developing pre-frailty, and the occurrence of three or more indicates frailty^2^.

The Brazilian older population is affected by individual and environmental factors, constituting a heterogeneous group composed of robust and frail older adults^2^. The latter is at higher risk of decreased functional capacity, falls, hospitalization, institutionalization, and early death^3^.

Frailty is associated with chronic diseases, such as hypertension and arthrosis^4^, and oral problems, such as dental caries and periodontitis, which are chronic multifactorial diseases^5^. The cumulative burden of these and other comorbidities can lead to tooth loss when not treated^4^. However, older adults do not perceive edentulism, caused by poor oral conditions for long periods, as harmful to their oral and general health. Based on aspects of daily life, this self-assessment is associated with the adaptation of diet, use of dentures, and continuity of satisfactory chewing^6^.

The absence of teeth is a critical factor in frailty, leading to changes in eating habits due to chewing problems^4^, which can reflect in difficulty chewing at least one type of food, including meats and raw fruits, thus demonstrating a deficiency in masticatory capacity^7^. Furthermore, edentulism and the use of dentures can be associated with greater psychosocial and functional aspects of chewing difficulties. Difficulty or pain when chewing hard food enhances changes in eating behaviors and consequent atrophy of the masticatory muscles, compromising the natural aging process^8^. However, despite these changes and their consequences for general health, the older adults perceive them as part of the natural aging process, self-reporting their oral health as excellent or good^9^.

The multicenter cohort study Health, Wellness and Aging (SABE) followed older adults for 10 years to, among other objectives, assess oral health and living conditions of this population, based on the individuals’ self-reported oral health measures and clinical evaluation^10^. The data generated were used to assess factors associated with tooth loss, showing that the older adults who used two removable dentures, men, lived alone, and self-assessed their oral health as regular or bad/very bad were at higher risk of tooth loss^11^. Based on these data, we could verify that the self-assessment of oral health is associated with the perception of the need for dentures. Moreover, the absence of functional dentition was associated with sociodemographic inequalities^12^.

However, there are few studies in developing countries demonstrating the relationship between the frailty of older adults and their self-perceived masticatory capacity. The study of this relationship is important for understanding the factors that affect older adults’ oral health and, thus, developing efficient public policies.

The FIBRA Project (Frailty in Older Brazilians) is a multicenter and multidisciplinary investigation dedicated to studying frailty in Brazilian older adults that can contribute to the assessment of the relationship between self-reported masticatory capacity and frailty at the contextual and individual levels of older people from six Brazilian municipalities^13^. Therefore, this study aims to investigate the relationship between masticatory function and frailty of community-dwellers older people. This study hypothesizes that the higher the number of alterations in the chewing function, the higher the likelihood of frailty in older individuals.

## METHODS

This exploratory cross-sectional study used secondary data from the FIBRA Study on the frailty conditions of older people living in urban areas of six Brazilian municipalities. The FIBRA Study aims to recognize the frailty conditions of older Brazilians based on biomarkers, social and psychological indicators related to chronic diseases, functional and cognitive impairment, oral health (including functional aspects of mastication), and other problems commonly associated with aging. This study was approved by the Human Research Ethics Committee of the School of Medical Sciences of the University of Campinas (No. 208/2007), by the Ethics Committee of the Piracicaba Dental School (No. 068/2015), and by Plataforma Brasil (No. 1,093,545 and CAAE: 45064815.7.0000.5418).

### Target Population

This study was composed of older men and women, aged 65 and over, living in six Brazilian municipalities: Campinas (SP), Belém (PA), Parnaíba (PI), Poços de Caldas (MG), sub-district of Ermelino Matarazzo (SP), and Ivotí (RS).

### Sampling Plan

Probabilistic samples of older adults from six Brazilian municipalities were selected, based on census tracts and home location, and considering male and female quotas proportional to the size of the older population in each municipality.

According to the sampling plan, the minimum sample size for Campinas and Belém (more than one million inhabitants) would be 601 older people (4% sampling error). For locations with less than one million inhabitants, the estimate was at least 384 older people (5% sampling error). In the municipality of Ivoti, where the population was smaller, all the older adults were counted (n = 646). The sample size was estimated considering a proportion in a finite population with α = 5%, 5% sampling error and 50% for the distribution of the variable under study, totalizing a sample of 235 older adults.

The exclusion criteria were: the presence of memory, attention, orientation, and communication issues suggestive of cognitive deficit, permanent or temporary inability to walk, localized loss of strength and aphasia due to stroke, severe impairment of speech, motor skills, or affectivity due to advanced Parkinson's disease, severe hearing or a visual impairment, and presence of terminal stage^2^. On the other hand, older adults aged 65 and over, of both sexes, owning their homes and in the census sector, without cognitive impairment, who were able to communicate and to understand instructions and who agreed to participate in the study were selected.

A total of 3,075 older people were recruited at their homes and subjected to a single data collection session divided into two parts. The first part included demographic and socioeconomic variables, anthropometric and frailty measures. If the participants scored over the cut-off point according to their schooling, they would participate in the second part of the protocol. Otherwise, the participant was dismissed. More information can be found in Neri et al., 2013^13^.

The cognitive screening used the Mini-Mental State Examination (MMSE), with cut-off scores suggested by the Brazilian Academy of Neurology based in Brucki et al.^14^ (minus one standard deviation). Regarding MMSE cut-off scores, “17” was used for illiterates; “22” for those with one to four years of study; “24” for those with five to eight; and “26” for those with nine or more. Different cut-off scores were chosen because cognitive deficits can affect the test reliability^2^.

Fried's criteria were adopted to classify frailty: unintentional weight loss, exhaustion/fatigue, handgrip strength (muscle weakness), physical activity level (sedentary lifestyle), and low gait speed. The older adults considered frail were those who met three or more of these criteria, pre-frail when they met one to two criteria, and non-frail (or robust) when they met any of the five established criteria^2^. The sample was later dichotomized into frail and non-frail, with the pre-frail and robust categories grouped.

### Data Collection

From September 2008 to June 2009, data collection took place in single sessions lasting from 40 to 120 minutes. After signing the informed consent form, MMSE was applied, followed by a questionnaire.

### Study Variables

Regarding the exposure variable masticatory function, an instrument consisting of 10 dichotomous questions (“yes” or “no”) was used: difficulty chewing and swallowing food, changes in palate or difficulty perceiving and differentiating flavors, difficulty or pain chewing hard foods, difficulty or pain swallowing, feeling of food stuck in the throat, food returning from throat to mouth, throat clearing after eating, gagging when eating or ingesting liquids, need liquids to help swallow food, and use of medicines to relieve toothache, proposed by Bellini (2006)^15^. Based on this instrument, a new variable was created considering the presence of “yes” answers, each “yes” representing a type of problem or difficulty in any of the functional aspects of mastication. Thus, this new variable was classified into: 0, 1-2 and 3 or more problems.

Other control variables were collected to verify the presence of an association between the outcome and the variable of interest (frailty and masticatory function), consisting of demographic and socioeconomic data, behavioral, general health, and oral health variables. A conceptual model was considered to assess the relationship of variables. This conceptual model considers frailty as being distally influenced by demographic and socioeconomic conditions, mesially by behavioral characteristics, and proximally by general health and oral health condition.

These variables were divided into three blocks:

• *Blocks 1 and 2 = Demographic and socioeconomic variables*: sex (male and female); age; ethnicity; personal income; schooling; family head (yes and no); and employed (yes and no).

The question “how old are you?” was used to collect the age, later dichotomized into 65–75 years old and 76 years or older. The self-reported ethnicity was stratified into four categories: White, Black, Brown/Mixed, and Others (Yellow, Oriental, and Indigenous). This last category contained only 1.9% of the individuals, none of them was frail, and therefore it was not possible to consider this variable in the analysis.

Personal income was collected according to minimum wages (MW) and dichotomized into less than or equal to one MW and more than one MW. As for schooling, this variable was obtained based on the following items: no formal education, 1 to 3, 4 to 8, 9 to 11, and 12 or more years of education. Subsequently, this variable was regrouped (up to three years and four years or more).

• *Blocks 3 and 4 = Behavioral and general health variables*: alcohol consumption (yes and no); smoking habit (yes and no); frequency of dental visits in the last 12 months (once or more and never); number of chronic diseases (up to one and two or more) that, according to the participant, were diagnosed by a physician in the year before the interview; depression (yes and no); and self-assessment of health compared to others of the same age (worse, equal, or better).

The number of chronic diseases variable considered the following health conditions: heart disease such as angina and heart attack; high blood pressure/ hypertension; stroke/ ischemia; diabetes mellitus; malignant tumor/ cancer; arthritis or rheumatism; lung disease such as bronchitis and emphysema, and osteoporosis. The presence of depression was assessed via the Geriatric Depression Scale (GDS-15) and considered a cut-off score greater than or equal to six^16^.

• *Block 5 = Oral health variables*: self-perceived dry mouth (in the last four weeks); presence of natural teeth; denture maladaptation (if it hurt or fall out); self-perceived oral health (bad/regular and good/excellent); and presence of sores on the tongue, cheek, palate or lip (if its presence is lasting more than one month). Exposure variable: “masticatory function.”

### Data Analysis

Stata 12 program was used for statistical analysis (Stata Corporation, College Station, TX). Odds ratio (OR), with its respective 95% confidence interval (95%CI), was the measure of adjustment between the individual factors and the outcome, estimated via multilevel logistic regression analysis using a fixed-effects model. This model allows for evaluating the possible effect of the explanatory variables on frailty, considering the sample structure, which was composed of older people living in six municipalities. Initially, the effect of the contextual variables, median nominal income, and literacy rate of the municipalities included in this study was evaluated. However, this analysis was not feasible due to the slight difference in the values observed for these variables between municipalities^17^.

After assessing the possible effects of the contextual variables, the multilevel logistic regression considered six adjusted models, organized hierarchically: the empty model included no factors; Model 1 included demographic variables; Model 2 included socioeconomic and demographic variables; Model 3 included behavioral, socioeconomic, and demographic variables; Model 4 included general health, behavioral, socioeconomic, and demographic variables, and the final model included the oral health variables adjusted for the ones above. These models only presented the OR of the variables that remained significant or adjusted the analysis. Thus, all associations were adjusted for the variables positioned in the same block and the preceding levels of the conceptual model, and the variables with p ≥ 0.05 were removed in sequence. The models were compared by verifying the log likelihood, which indicates the model quality of fit when inserting explanatory variables.

## RESULTS

Out of the 3,075 participants, 2,345 individuals (76.3%) did not have cognitive impairment. Due to lost to follow-up, the final sample size was 2,341 participants.

The mean age of the sample was 72.3 years old (± 5.48), and the mean number of functional mastication problems was 1.7 (± 1.83). According to Table 1, women (65.6%) and younger old adults (73.7%) were predominant. The majority (63.2%) reported not attending to a dentist last year. Approximately 26% of the sample had three or more functional feeding problems, and 7% (n = 164) were frail.

**Table 1:** Sample's characteristics. FIBRA, 2008–2009.

Characteristic	Category	n (%)
Demographic		
Sex	Female	1,537 (65.6)
	Male	806 (34.4)
Age (years old)	≥ 76	616 (26.3)
	65–75	1,726 (73.7)
Ethnicity	Other (Yellow/Oriental/Indigenous)	44 (1.9)
	White	1,310 (56.5)
	Black	200 (8.6)
	Brown/Mixed	765 (33.0)
Socioeconomic		
Personal income (MW)	≤ 1	1,073 (45.8)
	> 1	1,270 (54.2)
Currently works	No	1,942 (83.4)
	Yes	386 (16.6)
Education (years)	< 4	966 (41.3)
	≥ 4	1,374 (58.7)
Family head	No	849 (36.3)
	Yes	1,488 (63.67)
Behavioral		
Alcoholic consumption	Yes	707 (30.9)
	No	1,581 (69.1)
Smoker	Yes	205 (8.9)
	No	2,111 (91.1)
Frequency of dental visits in the last 12 months	Never	1,443 (63.2)
	≥ 1 time	840 (36.8)
General health		
Chronic diseases	≥ 2	1,530 (65.9)
	< 2	791 (34.1)
Depression – GDS	Yes	467 (22.1)
	No	1,643 (77.9)
Compared health assessment	Better	1,594 (70.2)
	Unchanged	526 (23.1)
	Worse	152 (6.7)
Oral Health		
Dry mouth	Yes	948 (41.3)
	No	1,350 (58.7)
Dentures hurt or fall out	Yes	459 (26.1)
	No	1,299 (73.9)
Natural teeth	No	1,072 (46.5)
	Yes	1,235 (53.5)
Oral health assessment	Bad/regular	680 (29.6)
	Good/great	1,616 (70.4)
Sore tongue, cheek, palate, or lip	Yes	158 (6.9)
	No	2,140 (93.1)
Masticatory function	0	772 (33.4)
	1–2	941 (40.7)
	3–10	598 (25.9)
Outcome		
Frailty	Frail	164 (7)
	Pre-frail/non-frail	2,179 (93)

GDS: Geriatric Depression Scale; MW: minimum wages.

Table 2 shows the association between self-assessment of oral health and functional aspects of mastication with frailty. All variables, except “choking on food or drinking liquids” and “using drugs to relieve toothache,” were related to frailty.

**Table 2:** Association of each of the ten criteria that compose the instrument functional aspects of mastication with frailty, FIBRA, Brazil, 2008–2009.

Characteristic		Non frail+ pre-frail	Frail	p
		n (%)	n (%)	
Masticatory function				
Difficulty chewing and swallowing hard food	Yes	154 (16)	236 (20.7)	< 0.001
Change in palate or difficulty perceiving and differentiating flavors	Yes	339 (15.8)	53 (32.7)	< 0.001
Difficulty or pain when chewing hard food	Yes	762 (35.5)	86 (53.4)	< 0.001
Difficulty or pain when swallowing	Yes	167 (7.8)	34 (21)	< 0.001
Feeling food stuck in the throat	Yes	387 (18)	53 (32.7)	< 0.001
Food returning from throat to mouth	Yes	191 (8.9)	24 (14.8)	0.013
Throat clearing after eating something	Yes	326 (15.2)	39 (24.1)	0.003
Choking on food or when drinking liquids	Yes	393 (18.3)	37 (22.8)	0.15
Need to drink liquids to help swallow food	Yes	404 (18.8)	61 (37.7)	< 0.001
Use of medicines to relieve toothache	Yes	108 (5.1)	08 (5)	0.97

The analysis in Table 2 also shows that more than half of the frail older population reported dry mouth (54%), being edentulous (56.8%), and difficulty or pain when chewing hard food (53.4%).

Table 3 shows the non-adjusted analysis of association. Older people with more unfavorable oral conditions, worse self-compared assessment of general health, worse socioeconomic conditions, and advanced age were more likely to be frail.

**Table 3:** Unadjusted (crude) analysis and odds ratio (OR) of the presence of frailty in older adults according to sociodemographic, behavioral, general health and oral health variables. FIBRA, Brazil, 2008–2009.

Characteristic	Category	Non-frail n (%)	Frail n (%)	OR	95%CI
Demographic					
Sex	Female	1,426 (65.44)	111 (67.68)	1.11	0.79–1.56
Age (years old)	≥ 76		78 (47.56)	2.76	2.00–3.82
Socioeconomic					
Personal income (MW)	≤ 1	985 (45.20)	88 (53.66)	1.38	0.10–1.90
Working	No	1,792 (82.77)	150 (92.02)	2.44	1.37–4.36
Schooling (years)	< 4	878 (40.33)	88 (53.99)	1.72	1.25–2.38
Family head	No	775 (35.65)	74 (45.40)	1.52	1.10–2.09
Behavioral					
Alcohol consumption	Yes	677 (31.75)	30 (19.23)	0.51	0.34–0.77
Smoker	Yes	185 (8.59)	20 (12.35)	1.54	0.94–2.52
Frequency of dental visits in the last 12 months	Never	1,329 (62.63)	114 (70.81)	1.40	0.98–2.00
General health					
Chronic diseases	≥ 2	1,406 (65.12)	124 (76.54)	1.76	1.21–2.56
Depression – GDS	Yes	395 (20.18)	72 (47.06)	3.52	2.51–4.94
Compared health assessment	Unchanged	488 (23.06)	38 (24.36)	1.42	0.95–2.11
	Worse	117 (5.53)	35 (22.44)	5.42	3.49–8.41
Oral Health					
Dry mouth	Yes	861 (40.29)	87 (54.04)	1.75	1.27–2.42
Denture hurt	Yes	410 (25.06)	49 (40.16)	2.01	1.38–2.94
Natural teeth	No	980 (45.69)	92 (56.79)	1.61	1.16–2.23
Oral health assessment	Bad/regular	613 (28.71)	67 (41.61)	1.77	1.27–2.46
Tongue sores	Yes	138 (6.46)	20 (12.42)	2.11	1.28–3.49
Mastictory function	1–2	882 (41.04)	59 (36.42)	1.70	1.08–2.69
	3–10	524 (24.38)	74 (45.68)	3.57	2.28–5.57

GDS: Geriatric Depression Scale; 95%CI: 95% confidence interval; MW: minimum wages.

In the final, adjusted model (Table 4), schooling and being head of household lost significance with the introduction of the behavioral variables. Older participants (OR = 2.88; 95%CI: 2.01–4.13), those who no longer work or were not active in the labor market (OR = 2.31; 95%CI: 1.18–5.53), those who were depressed (OR = 2.31; 95%CI: 1.58–3.38) and those who self-assessed their general health as worse compared to others of the same age (OR = 3.91; 95%CI: 2.39–6.40) had higher odds of being frail. Also, reporting three or more mastication problems was associated with a higher chance of frailty (OR = 2.06; 95%CI: 1.25–3.41), even after adjusting for age, work, depression, and oral health.

**Table 4:** Multilevel logistic regression of individual factors associated with frailty in older adults of six Brazilian municipalities. FIBRA, 2008–2009.

Characteristic		Empty model	Model 1	Model 2	Model 3	Model 4	Final model
Individual			OR (95%CI)	OR (95%CI)	OR (95%CI)	OR (95%CI)	OR (95%CI)
**Block 1: Demographic**							
Sex	Female		1.15 (0.81–1.62)				
Age (years old)	≥ 76		**2.78 (2.01–3.83)**	**2.53 (1.82–3.52)**	**2.69 (1.91–3.78)**	**2.89 (2.02–4.14)**	**2.88 (2.01–4.13)**
**Block 2: Socioeconomic**							
Personal income (MW)	≤ 1			1.05 (0.73–1.50)			
Working	No			**2.14 (1.16–3.94)**	**1.89 (1.02–3.49)**	**2.32 (1.19–4.55)**	**2.31 (1.18–4.53)**
Schooling (years)	< 4			**1.43 (1.02–2.01)**	1.37 (0.97–1.93)		
Family head	No			1.41 (0.99–2.00)	**1.42 (1.01–2.00)**		
**Block 3: Behavioral**							
Alcohol consumption	Yes				**0.62 (0.41–0.95)**		
Smoker	Yes				**1.79 (1.05–3.05)**		
Frequency of dental visits in the last 12 months	Never				1.22 (0.84–1.79)		
**Block 4: General health**							
Chronic diseases	≥ 2					1.25 (0.83–1.88)	
Depression – GDS	Yes					**2.63 (1.82–3.81)**	**2.31 (1.58–3.38)**
Compared health assessment	Unchanged					1.45 (0.95–2.21)	1.38 (0.90–2.11)
	Worse					**4.16 (2.54–6.80)**	**3.91 (2.39–6.40)**
**Block 5: Oral health**							
Functional aspects of mastication	1–2 problems						1.30 (0.79–2.14)
	3–10 problems						**2.06 (1.25–3.41)**
Intraclass correlation coeficient		0.008953	0.0088154	0.0110612	0.0151925	0.018907	0.0163313
**Log likelihood**		-593.42991	-574.70502	-556.64586	-520.91563	-468.34117	-464.0598

GDS: Geriatric Depression Scale; 95%CI: 95% confidence interval; OR: odds ratio; MW: minimum wages.

Note: Statistically significant values shown in bold.

## DISCUSSION

This study found an association between the presence of three or more problems related to self-assessed functional aspects of mastication and frailty among community-dwelling older adults. Since masticatory performance decline seems to be associated with worse general health status^18^, the development of frailty in older adults can be preceded by the loss of oral functionality caused by difficulty chewing, ingesting, and swallowing food, thus culminating in inadequate nutrition^19^ and affecting the quality of life. Our outcomes are corroborated by Lu et al.^20^ (2020) study, which deteriorated oral functions, such as dysphagia and decreased masticatory performance, contributed to poor oral health self-perception and quality of life. The literature describes vicious and harmful cycle of poor oral conditions and their effect on general health^21^. The lack of teeth compromises mastication and, consequently, decreases nutritional quality, triggering more health problems or leading to more severe conditions.

Studies have found that tooth loss compromises the masticatory function of older adults, changing eating habits, leading to a preference for soft foods that are easily chewed and swallowed^5,22,23^, usually poor in nutrients. Macro- and micro-nutrient deficiencies represent insufficient nutritional demands. This condition may lead to weight changes and fatigue^24,25^, declining strength and gait speed, the criteria included in the Frailty Phenotype. The impact of tooth loss, reported by 56.8% of the frail older adults in our study, is associated with limitations related to mastication and physical appearance, social life, and acute pain^26^. Such factors can influence older subjects’ behaviors and frailty development^27^.

The contextual covariates (city-level information on schooling and income) were not heterogeneous among the studied municipalities, thus suggesting contextual similarity (data not shown). The cross-sectional design of this is study is one of its limitations. The older adults participating in the FIBRA Study have already presented with extensive tooth loss, and it was not feasible to precisely establish when functional mastication problems began. Therefore, only the association between frailty and current oral health condition was indicated without inferring cause-and-effect relationships. However, studies suggest that dental prostheses have influenced injuries in this population, reducing individuals’ masticatory function, the latter being complex since it involves a bidirectional interaction with physical capacity. Additionally, we used a questionnaire to assess self-reported information on masticatory ability. This assessment does not allow to infer normative data on the masticatory performance. Also, cognitive decline is associated with aging and older adults that presented cognitive decline according to their schooling did not complete the second part of the protocol due to its self-reported nature, and it may represent a selection bias.

One of the main reasons for the relevance of this multicenter study is its contribution to understanding how oral health conditions and masticatory function are related to frailty, considering the lack of evidence in the literature on this topic, especially in Latin America. In addition, the study allows recognizing how older adults perceive their oral health condition, considering self-perception as a diagnostic tool that demonstrates the level of information of patients about their health condition. The instrument relates not only to the mastication ability but also assesses information that may be considered a proxy for dysphagia/presbyphagia. Dysphagia is defined as any disruption to the swallowing process, while presbyphagia relates swallowing changes with the aging process^20^. A study associates this condition with oral function and physical, cognitive, and psychological frailty^28^.

Our study found an association between the masticatory function and a greater chance of frailty among older participants. The examination of the dental condition cannot neglect the assessment of oral functions. Examinations should be part of the dental care routine, in order to help maintain a healthy oral and general status. Oral health services can contribute to prevent frailty in older subjects by expanding the examination of denture quality and fitting, dry mouth, and mucosal sours in older subjects. Oral health promotion can prevent frailty and contribute to the reversibility of frailty if already established. Therefore, the evaluation of self-perceived masticatory function should be included as part of frailty screening and treatment, considering the inclusion of oral health professionals into multidisciplinary teams to deliver integrated comprehensive patient care.
